# Strength Training Session Induces Important Changes on Physiological, Immunological, and Inflammatory Biomarkers

**DOI:** 10.1155/2018/9675216

**Published:** 2018-06-26

**Authors:** Ayla Karine Fortunato, Washington Martins Pontes, Débora Maria Soares De Souza, Jéssica Santos Ferreira Prazeres, Lucas Soares Marcucci-Barbosa, Júlia Miranda Mól Santos, Érica Leandro Marciano Veira, Eduardo Bearzoti, Kelerson Mauro De Castro Pinto, André Talvani, Albená Nunes Da Silva

**Affiliations:** ^1^Laboratório de Inflamação e Imunologia do Exercício (LABIIEX), Centro Desportivo da Universidade Federal de Ouro Preto (CEDUFOP/UFOP), Ouro Preto, MG, Brazil; ^2^Laboratório da Imunobiologia da Inflamação (LABIIN/ICEB), Instituto de Ciência Exatas e Biológicas (ICEB/UFOP), Ouro Preto, MG, Brazil; ^3^Laboratório Interdisciplinar de Investigação Médica (LIIM), Faculdade de Medicina (UFMG), Belo Horizonte, MG, Brazil; ^4^Departamento de Estatística, Universidade Federal de Ouro Preto (UFOP), Ouro Preto, MG, Brazil

## Abstract

Strength exercise is a strategy applied in sports and physical training processes. It may induce skeletal muscle hypertrophy. The hypertrophy is dependent on the eccentric muscle actions and on the inflammatory response. Here, we evaluate the physiological, immunological, and inflammatory responses induced by a session of strength training with a focus on predominance of the eccentric muscle actions. Twenty volunteers were separated into two groups: the untrained group (UTG) and the trained group (TG). Both groups hold 4 sets of leg press, knee extensor, and leg curl at 65% of personal one-repetition maximum (1RM), 90 s of recovery, and 2^″^conc/3^″^eccen of duration of execution in each repetition. Blood samples were collected immediately before and after, 2 hours after, and 24 h after the end of the exercise session. The single session of strength training elevated the heart rate (HR), rating of perceived exertion (RPE), visual analog scale (VAS), and lactate blood level in UTG and TG. Creatine kinase (CK) levels were higher at 2 and 24 h after the end of the exercise in UTG and, in TG, only at 24 h. The number of white blood cells (WBC) and neutrophils increased in UTG and TG, post and 2 h after exercise. Lymphocytes increased postexercise but reduced 2 h after exercise in both groups, while the number of monocytes increased only immediately after the exercise session in UTG and TG. The strength training session elevated the levels of apelin and fatty acid-binding proteins-3 (FABP3) in both groups and brain-derived neurotrophic factor (BDNF) in TG. The single exercise session was capable of inducing elevated HR, RPE, lactate level, and CK levels. This protocol changed the count/total number of circulating immune cells in both groups (UTG and TG) and also increased the level of plasmatic apelin, BDNF, and FLTS1 only in TG and FABP3 myokines in both groups.

## 1. Introduction

Regular physical exercise has achieved wide acceptance by the overall population, professional organizations, and the medical community. Many important international communities—such as UNESCO and ACSM—have been stimulating the inclusion of regular physical exercise for the population around the world. Nowadays, there is a strong body of scientific evidences for prescribing physical exercise as a prevention and therapy in many different chronic diseases. This amount of information suggests that physical exercise is able to work as a therapy on these disease pathogenesis and symptoms, and the interpretation of these scientific literatures can also indicate the optimal type and dose for prescription of physical exercise [1–3]. Regular physical exercise can be driven by different protocols, such as aerobic, anaerobic, endurance, strength, or flexibility and—with their particularities—can promote reducing risk of obesity and metabolic syndrome-associated diseases, as well as benefits in morph functional alterations in the body [4]. According to the adjustment of the exercise protocol, it can cause temporary microtraumas of varying degrees in skeletal muscles [5, 6]. These skeletal muscle microtraumas induce the tissue regeneration process. In this process, immune cells such as neutrophils and macrophages are activated to work in the recovery of tissue homeostasis, producing pro- and anti-inflammatory mediators (IL-6, TNF-*α*, and IL-10) [7–10]. In addition, the microtraumas which are exercise-induced are dependent on the load components and include disruption of the extracellular matrix and basal lamina of the sarcolemma. This may result in the release into the blood, intracellular proteins such as myoglobin (Mb), lactate dehydrogenase (LDH) and aspartate aminotransferase, and creatine kinase (CK) [6].

The protocols of strength training exercises are an essential part of various training processes, such as in health maintenance, in recreational practitioners, and in the improvement of professional sports performance [11–14]. Strength exercise protocols are classified as predominantly anaerobic due to their characteristics (intensity and duration) and allow greater control of muscle actions (concentric, eccentric, and isometric) [15, 16].

The strength training has been related to the induction of skeletal muscle hypertrophy according to the following parameters: load components around 60–85% of the values obtained in maximum strength test (1RM (one repetition maximum)), three to six series, six to twelve repetitions, and pauses between sets of one to three minutes [15].

Since there is a close relation between exercises with the systemic and local inflammatory response [7–10], inflammatory exercise-induced processes emerge as beneficial and necessary processes, as once it is a mechanism responsible for the regeneration and repair of skeletal muscle tissue. However, the inflammatory process which is exercise-induced needs to be maintained under control to be efficient. Situations, including type of exercise, amount of muscle recruited, and type of muscle action (concentric and eccentric), define the magnitude of local and systemic inflammation. Another important aspect may be the status of practitioners.

There is a body of evidence showing that many types of exercise protocols can modulate the plasma level of myokines, which are cytokines produced in the skeletal muscle tissue. The entire “secretome” of exercising skeletal muscle has not yet been described. Here, we investigated some myokines such as apelin, brain-derived neurotrophic factor (BDNF), fatty acid-binding proteins-3 (FABP3), follistatin-like-1 (FLST1), osteonectin, and interleukin-15 (IL-15). Apelin is important in increasing the strength of cardiac contraction [17] and is also associated with insulin metabolism. BDNF when produced and secreted in muscle tissue has metabolic properties increasing fat oxidation by AMPK activation, and this molecule also regulates satellite cell differentiation and regeneration of skeletal muscle tissue [18]. FABP3 is a protein that facilitates the transport of intracellular fatty acids, and chronic physical exercise is effective in provoking an upregulation, since trained individuals have a greater expression of this molecule [19]. FLST1 exerts therapeutic effects by modulating cardiac hypertrophy in heart failure (HF) with *preserved* ejection fraction (HFpEF) [20]. Recently, many studies have attributed exercise as a cancer-inhibiting factor, but the mechanisms remain unclear. Osteonectin is a protein with the capacity to influence pathways involved in extracellular matrix assembly such as procollagen processing and collagen fibril formation as well as the capacity to influence osteoblast differentiation and osteoclast activity [21]. Interleukin-15 (IL-15) has been considered an anabolic myokine due to its presence when promoting the synthesis of contractile proteins [22].

In this sense, the aim of this study was to investigate the effects of single strength training sessions on physiological (HR, RPE, VAS, and lactate), immunological (leukocyte number), and skeletal muscle mediators, for example, myokines, apelin, BDNF, FABP3, FLST1, osteonectin, and IL-15, in trained (TG) and untrained (UTG) subjects.

## 2. Materials and Methods

### 2.1. Subjects

Twenty young male volunteers ranging from 18 to 35 years old were separated into two groups: an untrained group (UTG), weight of 74.8 ± 14.2 (kg) and a trained group (TG) consisting of trained practitioners, weight of 72.2 ± 3.8 (kg), that had been practicing strength training for at least 6 months continuously. The inclusion criteria for both groups were the absence of musculoskeletal lesions in the last six months in the lower limbs, spine, and pelvis; no smoking; no drinking of alcohol for at least 3 days prior to the study. The exclusion criteria for the volunteers were absence on the test day, any disease and/or clinial condition that compromises the performance, or any use of anabolic hormones or supplements. Ethical clearance for this study was obtained from the Ethical Committee of the Federal University of Ouro Preto, MG (Res. 196/96 - CAAE 56307716.2.0000.5150).

### 2.2. Strength Exercise Protocol

The volunteers performed a strength training session according to the load regulations for skeletal muscle hypertrophy. Immediately before, immediately after, and 2 hours after the training session, blood samples were collected from the radial vein to quantify physiological markers, leukocytes, and myokines. All procedures were performed in the Laboratory of Inflammation and Exercise Immunology (LABIIEX/CEDUFOP) from Ouro Preto University (UFOP), Ouro Preto, Brazil.

Each volunteer came to the laboratory for a total of three times. On the first day, the volunteers were submitted to a physical examination which determined their body composition (body fat), weight, height, and circumference of the thigh and calf, as well as determining the range of motion of the knee joint. On the second trial day, the volunteers performed the one-repetition maximum test (1RM), and on the third trial day—respecting a minimum interval of one week (7 days)—the volunteers performed a single strength training session in a leg press, knee extensor, and leg curl. First, the volunteers warmed up in a cycle ergometer for 5 minutes in a low intensity. After that, both groups (UTG and TG) performed 4 sets of leg press, knee extensor, and leg curl exercises in this sequence. The overload was adjusted at 65% of personal one-repetition maximum (1RM) for each machine. The time of recovery was 90 s, and the duration of muscle tension was 2 seconds for concentric action and 3 seconds for eccentric action in each repetition. The volunteers performed between 8 and 10 repetitions for each exercise. All training sessions spent around 35–40 minutes. The volunteers were oriented not to perform any physical exercise at least for 3 days. After the training session, peripheral blood was collected immediately before, immediately after, 2 hours after, and 24 hours after the end of exercise. Blood samples were collected from the median cubital vein by a professional nurse. The collected blood was taken to the Immunobiology of Inflammation Laboratory (LABIIN), Clinical Analysis Laboratory (LAPAC), and Interdisciplinary Laboratory of Medical Investigation (LIIM) to be analyzed. The blood samples were taken from the median cubital vein in the forearm using two different types of tube: an S-Monovette® tube 2.7 ml, EDTA K3, was used for hemogram analyses, and an S-Monovette 7.5 ml Serum tube that does not contain any anticoagulant was used for plasma separation for protein analyses. The blood was transported using standard conditions for transport of biological materials. The time spent to transport the blood samples was around 10 minutes in appropriate conditions in a cooler box with ice.

### 2.3. Heart Rate (HR), Rate of Perceived Exertion (RPE), and Visual Analog Scale (VAS)

Heart rate was assessed by using a personal POLAR tool, and the values were recorded during the all-strength training session. After each set of exercise for every session, the volunteers noted their rate of perceived exertion using the Borg scale [23] as modified by Robertson et al. [24] (modified 0–10 Borg scale/OMNI-RES) to summarize the total perception of physiologic stress in the whole body, and the visual analog scale (VAS) [25] was used to represent the perception of specific pain in the legs.

### 2.4. Lactate Level

The lactate level for peripheral blood circulation was analyzed by using Accutrend Plus (Roche) before, immediately after, and 2 hours after the end of the strength training session.

### 2.5. Postworkout Nutrition Strategy

To prevent volunteers from suffering from dehydration, fatigue, and sudden drop of glucose, they were fed within 30 minutes of posttraining. The meal was based on the mean age of the groups (25.5 years) and weight (73.45 kg), using the FAO/WHO formula (1985) for energy determination (15.3 × P + 679), plus 30% of the total value, since the activity was preformed until exhaustion. The average daily caloric recommendation for volunteers was 2.343 kcal/day, with 20% dedicated to the posttraining meal. The diet followed the recommendation of the Brazilian Society of Sports Medicine (2009) and focused on the supply of complex carbohydrates (about 70% of the total amount of calories offered), since it plays a crucial role in energy supply and—after the effort carbohydrate intake—aims to restore depleted glycogen stores, to guarantee anabolic standard, and to reduce protein degradation [26–28].

### 2.6. Full Blood Counts (Hemogram)

Full blood counts were performed using a five-part differential hematology analyzer (Beckman Coulter AcT 5diff AL Hematology Analyzer, California, USA). The hematology analyzer uses a sequential dilution system and dual-focused flow fluid dynamic technologies employing the Coulter principle of impedance to count and size the cells.

### 2.7. Human Myokine Protocol

In this study, a HMYOMAG-56K MILLIPLEX® MAP Human Myokine Magnetic Bead Panel and Luminex® were used for analysis of myokines following the manufacturer's protocols. In this study, the human myokines analyzed were apelin, brain-derived neurotrophic factor (BDNF), fatty acid-binding proteins-3 (FABP3), follistatin-like-1 (FLST1), osteonectin, and interleukin-15 (IL-15).

### 2.8. Statistical Analysis

Initially, characterization traits (weight, height, percentage of body fat, and age) of both groups (TG and UTG) were compared with *t*-tests. Statistical models were then fitted, according to the factors of group, time, and their interaction, using PROC MIXED of the SAS software [29]. Firstly, the model of a split plot design in time was considered. However, since time cannot be randomized, there may be a covariance structure for the observations other than the one ordinarily induced by the randomization procedure [29]. Therefore, an alternative and more general model was also considered, with an unstructured residual covariance structure, using the option *type=un* in the *repeated* statement [30]. Such models were compared according to the Akaike criterion, and in all cases, the split plot model provided a better fit. The factors group, time, and their interaction were tested using type 3 *F* tests, with a significance level of 0.05. If the null hypothesis of such tests was rejected, the least squares means were obtained, which is more suitable for unbalanced data, as in the present study, where the final number of volunteers was different in each group. Least squares means of factor time were compared using the Tukey *post hoc* adjustment. The assumption of normal distribution for the residuals of the fitted models was verified with the Shapiro-Wilk test. If this assumption was violated, the level combinations of groups and times were compared with the nonparametric Kruskal-Wallis test. In case of significant differences, Dunn's multiple comparison test was used to identify where these differences resided. Nonparametric analyses were used with the Prism software. To correlate lactate level and leukocytes, the Pearson correlation analysis with linear regression was used. The level of significance is 95% (*p* = 0.05).

## 3. Results

The volunteers' weight was 74.8 ± 14.2 (kg) in UTG, while in TG it was 72.2 ± 3.8 (kg). The mean of height in UTG was 175.1 ± 8.4 (cm) and, in TG, 173.5 ± 7.7 (cm), while the percentage of body fat (% BF) was 15.0 ± 8.7 and 9.8 ± 2.9 in UTG and TG, respectively. The mean of age (years) of the volunteers in UTG was 24.5 ± 2.8 and in TG 26.6 ± 1.3. There was no difference (*p* = 0.10) in a % BF values between TG and UTG volunteers (Table 1).

Table 2. Characterization of volunteers. This table shows the absolute values and means of each volunteer for weight (kg), height (cm), percentage of body fat (%BF) and age (years) (*p* = 0.05).

### 3.1. Strength Training Session Induces Physiological Changes

**Figure 1:**
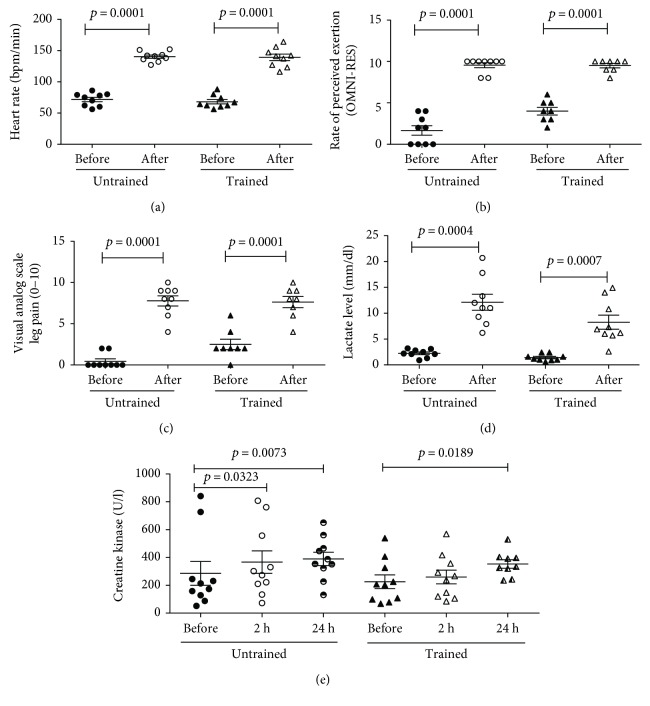
The strength training exercise protocol alters physiological markers. The single strength training protocol session elevated the heart rate in UTG and TG. The RPE and VAS were also elevated in both groups. The lactate levels were elevated differently when comparing UTG and TG. The CK level increased at 2 h and at 24 hours in UTG and in TG only 24 hours after the end of exercise. Data are presented in mean and standard errors, and the level of significance is *p* < 0.05.

After the single strength training protocol session, the heart rate increased from 71.8 ± 10.4 to 140.2 ± 8.2 bpm/min (*p* < 0.0001) in UTG and from 68.1 ± 10.5 to 139 ± 15.5 bpm/min (*p* < 0.0001) in TG (Figure 1(a)), and the rate of perceived exertion (RPE/OMNI-RES) was elevated from 1.7 ± 1.7 to 9.6 ± 0.9 (*p* < 0.0001) in UTG and from 4.0 ± 1.3 to 9.5 ± 0.8 (*p* < 0.0001) in TG (Figure 1(b)). In the same direction, the visual analog scale (VAS) was elevated from 0.4 ± 0.9 (*p* < 0.0001) in UTG and from 2.5 ± 1.8 to 7.6 ± 1.9 (*p* < 0.0001) in TG (Figure 1(c)). The lactate levels were elevated from 2.2 ± 0.8 to 12.1 ± 4.6 mmol/dl (*p* < 0.0004) in UTG, and interestingly, TG had a lower elevation from 1.4 ± 0.6 to 8.3 ± 4.1 mmol/dl (*p* < 0.0007), and it was statistically different (Figure 1(d)). Furthermore, the level of circulating creatine kinase (CK) increased from 286 ± 270.7 (U/l) for the pre-exercise situation to 367 ± 255 (U/l) at 2 h and 389 ± 151 (U/l) at 24 hours in UTG and in TG from 225 ± 155 (U/l) for the pre-exercise situation to 353 ± 90 (U/L) at 24 hours after the end of exercise (Figure 1(e)).

### 3.2. A Single, Lower Member Strength Training Session Was Able to Change the Count of White Blood Cells

**Figure 2:**
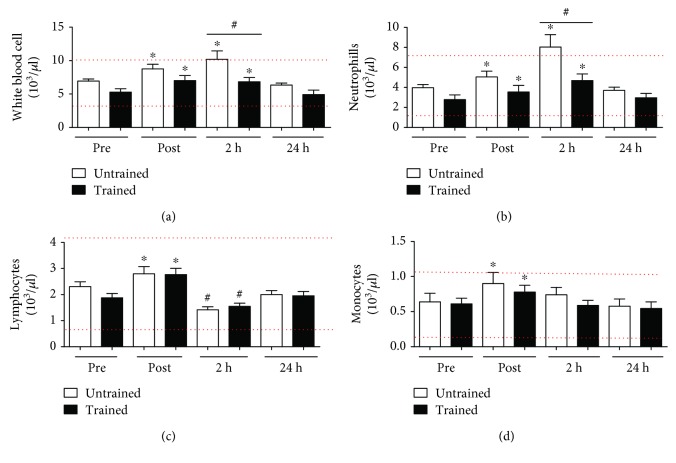
The strength training exercise protocol alters the number of subpopulation in the white blood cells (leukocytes). The red lines mean the reference value. The single session of exercise was able to elevate the number of white blood cells immediately and 2 hours in UTG and TG (Figure 2(a)). The number of neutrophils was elevated differently in both groups (Figure 2(b)). The number of lymphocytes increased immediately after in both groups and then reduced at 2 h (Figure 2(c)). The monocyte number increased in both groups (Figure 2(d)). Data are presented in least squares means and standard error, with the level of significance adopted at *p* < 0.05. ^∗^Difference between time points and ^#^difference between groups.

The strength training exercise protocol increased the number of white blood cells from 6.9 ± 0.9 (10^3^/*μ*l) to 8.7 ± 2.1 (10^3^/*μ*l) immediately after and 10.2 ± 3.9 (10^3^/*μ*l) at 2 hours after exercise in UTG, and from 5.2 ± 1.5 (10^3^/*μ*l) to 7.0 ± 2.3 (10^3^/*μ*l) and 6.8 ± 1.9 (10^3^/*μ*l) in the peripheral blood circulation in TG (Figure 2(a)) (*p* < 0.0001). The data shows an increase in the number of circulating neutrophils from 3.9 ± 1.0 (10^3^/*μ*l) to 5.0 ± 1.8 (10^3^/*μ*l) immediately after and 8.0 ± 3.9 (10^3^/*μ*l) at 2 hours after the end of the session in UTG (*p* < 0.0001). The exercise protocol also elevated the number of neutrophils from 2.7 ± 1.4 (10^3^/*μ*l) to 3.5 ± 2.0 (10^3^/*μ*l) immediately after and 4.6 ± 2.0 (10^3^/*μ*l) at 2 h after the end of the session in TG (Figure 2(b)) (*p* < 0.0001). Lymphocyte cells also increased from 2.3 ± 0.5 (10^3^/*μ*l) to 2.8 ± 0.8 (10^3^/*μ*l) in the moment immediately after in UTG and from 1.8 ± 0.5 (10^3^/*μ*l) to 2.7 ± 0.7 (10^3^/*μ*l) in TG (*p* < 0.0001). Interestingly, the number of lymphocytes reduced in both groups to 1.4 ± 0.3 (10^3^/*μ*l) in UTG and to 1.5 ± 0.3 (10^3^/*μ*l) in TG at 2 hours after the end of the exercise session (Figure 2(c)) (*p* = 0.0004). In addition, the number of monocytes increased from 0.6 ± 0.3 (10^3^/*μ*l) to 0.9 ± 0.4 (10^3^/*μ*l) in UTG and from 0.6 ± 0.2 (10^3^/*μ*l) to 0.7 ± 0.3 (10^3^/*μ*l) only immediately after the end of the strength exercise protocol (Figure 2(d)) (*p* < 0.0001). The elevation of the subpopulations of leukocytes had a different behavior after the strength training exercise protocol when comparing UTG and TG.

### 3.3. There Is a Correlation between the Intensity of Exercise and the Perturbation of the Immune System

**Figure 3:**
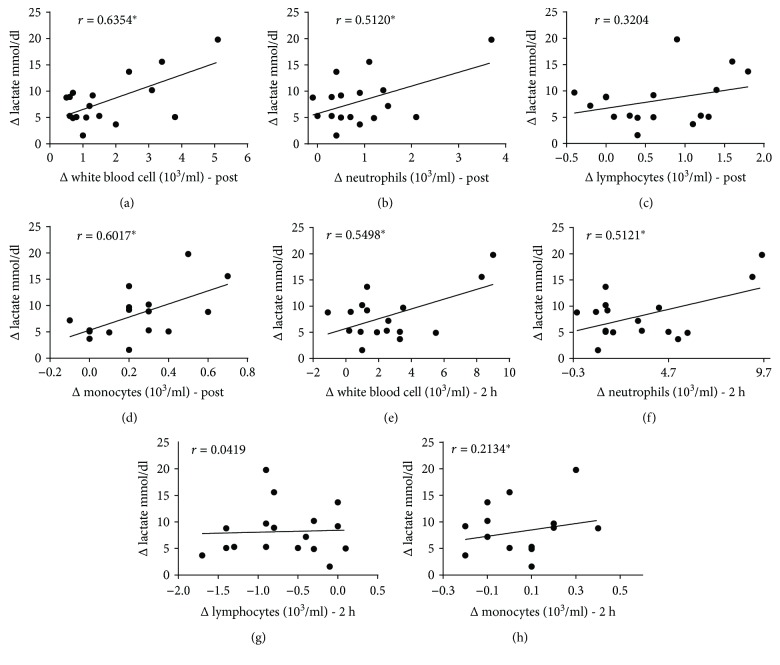
The plasma level of lactate was positively correlated with the increase in the number of leukocytes. There was a relationship between the plasma lactate level and the number of total leukocytes (Figure 3(a)), neutrophils (Figure 3(b)), and monocytes (Figure 3(c)) immediately after exercise. This relationship is still maintained with leukocytes (Figure 3(e)) and neutrophils (Figure 3(f)) 2 hours after the end of the session. ^∗^Statistical significance.

The plasma lactate level positive correlated with the total number of leukocytes (Figure 3(a)), neutrophils (Figure 3(b)), and monocytes (Figure 3(c)) immediately after exercise. This parameter also directly correlated with total number of leukocytes (Figure 3(e)) and neutrophils (Figure 3(f)) 2 hours after the end of the exercise session.

### 3.4. The Strength Training Exercise Protocol Was Able to Elevate the Myokine Levels in the Circulation

**Figure 4:**
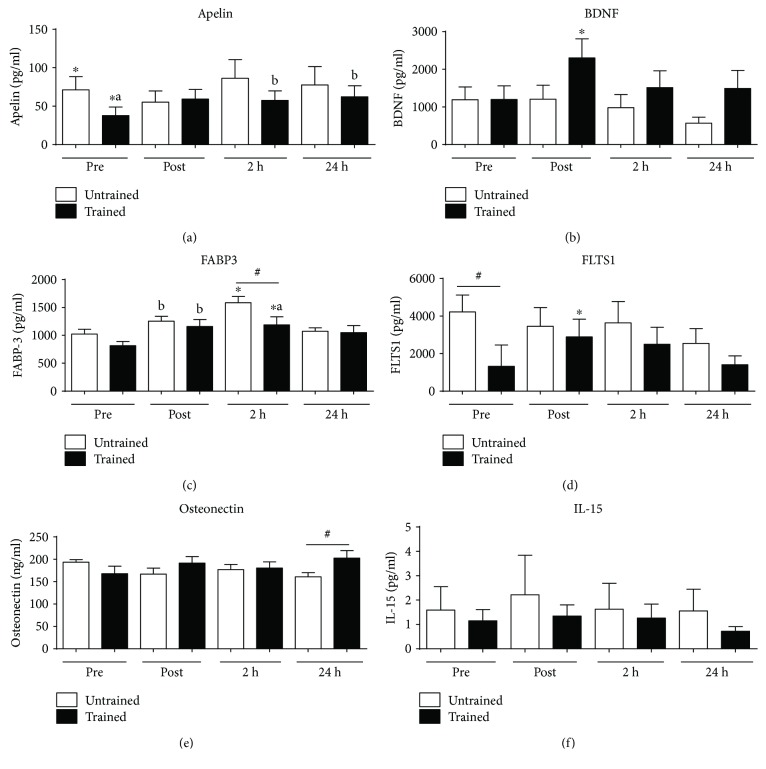
The single strength training exercise protocol induces alterations in the level of circulating myokines. The single session of exercise was able to elevate the level of some myokines IL-6 in TG 2 hours (Figure 3(a)), BDNF in UTG 2 hours (Figure 3(c)), and FABP3 immediately after exercise and 2 hours (Figure 3(g)). This exercise protocol did not change IL-15 (Figure 3(b)), apelin (Figure 3(d)), osteonectin (Figure 3(f)), and oncostatin (Figure 3(h)). Data are presented in least squares means and standard error, with the level of significance adopted at *p* < 0.05. ^∗^^,^^#^^, a^^, b^ Statistical difference between groups.

The strength exercise protocol was able to change the levels of important myokines. The plasmatic apelin levels increased in UTG (Figure 4(a)) 2 h and 24 h after the exercise protocol (*p* = 0.0025). The BDNF levels increase only in TG immediately after the end of exercise (Figure 4(b)) (*p* = 0.0147). The FABBP3 molecule levels increased in both groups (UTG and TG) postexercise and 24 hours after the end of the session (Figure 4(c)) (*p* < 0.0001). FLTS1 increased postexercise only in TG (Figure 4(d)) (*p* = 0.0467). FLTS1 (Figure 4(d)) expressed different levels (*p* = 0.0293) when comparing the basal level between TG and UTG. The single strength training session had no difference in the plasmatic levels of oncostatin (Figure 4(e)) and IL-15 (Figure 4(f)) molecules.

## 4. Discussion

There are some noteworthy findings from this study: (i) the single strength training session was able to induce physiological stress, (ii) the local skeletal muscle contraction (lower members) was able to change the leukocyte counting in the peripheral blood, and finally (iii) the single strength training session is an enough stimuli to produce and release myokines (apelin, BDNF, FABP3, and FLTS1) in the peripheral blood circulation.

The monitoring of the physiological workload in this protocol was guaranteed by the necessity of stimuli modifying the body homeostasis. The strength training exercise protocol was able to change physiological parameters such as heart rate (HR), rate of perceived exertion (RPE), and visual analog scale (VAS). In addition, this strength exercise protocol also increased the level of lactate (immediately after) and creatine kinase (2 and 24 hours) in the blood circulation from the volunteers. The interesting result here is that the level of increase in lactate levels immediately after the end of exercise was different when comparing UTG and TG. TG appears to produce less lactate or may remove it faster than does UTG from the circulation. The possible explanation for this perception could be the increase in key enzyme activities induced by the training process, such as enzymes of glycolysis, such as glycogen phosphorylase, phosphofructokinase (PFK), and lactate dehydrogenase (LDH) [31]. Herein, in this study, the plasmatic level of CK increased in both TG and UTG; however, in TG this increase occurred only 24 h after the end of the strength exercise session. The appearance of CK in blood has been generally considered to be an indirect marker of muscle damage, and unaccustomed exercise, particularly eccentric muscle contractions, initiates mechanical muscle damage of varying degrees [6].

Over the past two decades, a variety of studies has demonstrated that exercise induces considerable physiological change in the immune system. Acute and chronic exercise alters the number and function of circulating cells of the innate immune system (e.g., neutrophils, monocytes, and natural killer (NK) cells) [32, 33]. Acute physical exercise is able to change immunological variables up to 8 hours postexercise, including significant NK cell suppression, NK cell phenotype changes, a significant increase in total lymphocyte counts, and a significant increase in eosinophil cell counts all at 8 hours postexercise [34]. High intense interval training (HIIT) is able to promote lymphocyte oxidative stress and reduce super antigen-induced proliferation [35]. Long-distance running is able to increase the number of circulating total leukocytes with the increase in neutrophils, monocytes, and lymphocytes including B and T cells [36]. Therefore, it has been suggested that exercise represents a physical stress that is able to modify the counting of immune cells in the peripheral blood circulation. The elevation in the number of the white blood cells, neutrophils, lymphocytes, and monocytes was investigated in cycling [34, 37], treadmill [38–41], running race [42–44], eccentric unilateral repetitions of knee extensors [45], aerobic exercises, such as gymnastics and circular dance [46], and four different experimental conditions [47].

However, there is less information about the effects of the strength training protocol on the response of number of white blood cells and in the levels of myokines. The analyses of results show that the single session of strength training for lower members can elevate the total number of white blood cells. In UTG, the number of white blood cells was increased immediately after, had a further increase 2 hours after the end of exercise, and then returned to basal levels 24 hours after the end of the session. In UTG, the number of white blood cells was equally higher immediately and 2 hours after the end of exercise (Figure 2(a)). This elevation in the number of white blood cells was followed by the increase in the number of neutrophils postexercise and further increased 2 hours after the end of the exercise session in TG and UTG (Figure 2(b)). The lymphocyte number also increased immediately after exercise and then expressed an important reduction in the number of these cells in both UTG and TG (Figure 2(c)). This lymphopenia phenomenon was previously described after the end of different exercise protocols and could be associated to the “The open window theory” that is characterized by short-term suppression of the immune system following an acute bout of endurance exercise. The biphasic response of total lymphocyte numbers may be due to circulating stress hormones, including epinephrine, norepinephrine, and cortisol. It has been suggested that catecholamines induce the initial increase in lymphocyte number [34]. Another important aspect of this data is that the status of physical condition may change the response to the same relative physical stress when comparing TG and UTG. There are a robust number of papers showing changes in the number of white blood and subpopulation cells after different exercise protocols; however, there is less information about the effects of single session for strength training on the number of peripheral blood cells [34, 48, 49]. The elevation in the number of leukocytes after a single strength training session could be related to the role of these cells in the regeneration of skeletal muscle tissue, as this cell may transmigrate the endothelial barrier and work to regenerate the skeletal muscle tissue that was damaged during the exercise [50, 51].

Interestingly, the plasma level of lactate was positively correlated with the increase in the number of total leukocytes, neutrophils, and monocytes immediately after exercise and with leukocytes and neutrophils 2 hours after the end of the session (Figure 3). These data show that the immune system is responsive to the intensity of exercise in a strength training protocol. The important question about the relationship between exercise and immune system is the possibility of “training” (modulate) the immune response by the physical exercise intensity. Furthermore, the effects or intensity and duration of exercise on this modulation of immune system response are completely unknown. The meaning of the correlations between the intensity of exercise marked by the lactate levels and the number of leukocytes could show that the more intense the exercise session is, the more stress it can induce in the immune system. However, there was no relationship between intensity and any myokines (data not shown).

This process seems to produce some of the symptoms associated with muscle injury, including the loss of muscle function, delayed onset muscle soreness (DOMS), and an increase in muscle proteins in circulation representing damage to the plasma membrane [10]. After injury, the repair mechanism of the muscle damage is highly synchronized, and this inflammatory response needs to be controlled; otherwise, the inflammatory cells could cause extra damage by producing reactive oxygen species (ROS) and local proinflammatory cytokines [8, 49]. Therefore, this local inflammatory process needs to be maintained under careful control to recover homeostasis and skeletal muscle tissue function.

Studies involving systemic inflammatory responses following exercise protocols have demonstrated a variety of responses, such as the type of exercise, the amount of muscle tissue recruited, the status (aerobic or anaerobic), the type of muscle action (concentric or eccentric), and lastly, the duration and intensity which define the magnitude of local and systemic inflammation [6, 52]. It is known, for example, that the number of circulating leukocytes increases after a “downhill” and eccentric bicycle exercise [34–37]. In contrast, there is a minor variation in plasma concentrations of circulating leukocytes after two kinds of exercise—eccentric quadriceps and flexor elbow [53–55].

Cytokines are responsible for the coordination, amplification, and regulation of the magnitude and duration of inflammatory events and their effects [56–58]. Skeletal muscle can produce cytokines, and in this scenario, they are called myokines [1–3]. After the intensive physical exercise of force, and possible release of cytokines into the bloodstream, there is an increase in the influx of neutrophils, monocytes, lymphocytes, and other cells that participate in tissue regeneration, indirectly signaling the increased permeability of blood vessels and thus an increase in the transition of fluid and proteins into the extracellular space [59]. Cytokines are produced and released primarily by cells of the immune system, in addition to the active muscle cells, adipose tissue, and endothelial cells, and have proinflammatory (IL-1*β*, TNF-*α*, and IL-6) and regulatory (IL-6, IL-10, and IL-1ra) modulation [60]. The balance between proinflammatory and regulatory actions of the various cytokines contributes to the complete regeneration of damaged tissue [7–9].

Variations in inflammatory responses of strength exercises are related to the duration, intensity, and other physiological factors related to stress, such as body temperature, endocrine response, metabolic acidosis, oxidative stress, and muscle damage caused by the exercise session [6, 32, 58]. According to some authors, a reduced inflammatory response would also be accompanied by a reduced regenerative capacity. It is now firmly established that skeletal muscle tissue produces and secretes cytokines and other proteins, which have been named “myokines,” and these proteins subsequently exert auto-, para-, and/or endocrine effects. Thus, the skeletal muscle can be classified as an endocrine organ [1–3]. Since the description of the first members, the list of myokines has constantly been growing demonstrating that the skeletal muscle has the capacity to express several myokines—some simultaneously, others in a temporally or context-controlled manner. For most currently described myokines, contractile activity is the key regulatory element for expression and secretion.

The finding that the muscle secretome, which is an endocrine organ, consists of several hundred secreted peptides provides a conceptual basis and a whole new paradigm for understanding how muscles communicate with other organs, such as the adipose tissue, the liver, the pancreas, the bones, and the brain [1–3]. The analyses of the results show that a single session of strength training is able to modulate some of myokines.

The basal plasma of UTG and TG volunteers shows different levels of apelin (Figure 4(a)), a potent inotropic agent and vasodilator molecule, and this result can indicate that the status training can influence the level of circulating cytokines [61]. The strength training session increases the level of myokines at 2 h and 24 h in TG. This molecule is widely expressed in various organs such as the heart, lung, kidney, liver, adipose tissue, gastrointestinal tract, brain, adrenal glands, endothelium, and human plasma and is associated with pressure control, cardiac embryogenic development, and differentiation of adipose tissue.

Apelin is important in increasing the strength of cardiac contraction [17] and is also associated with insulin metabolism. BDNF is a neurotrophic factor also produced by skeletal muscle cells. BDNF acts on certain neurons of the central nervous system and the peripheral nervous system, helping to support the survival of existing neurons and encouraging the growth and differentiation of new neurons and synapses. A single session of strength training was able to increase the level of BDNF in TG when comparing the time before and immediately after the session (Figure 4(b)). The increase in BDNF after exercise has been described and could be associated with the protection to the nervous system against Alzheimer's, Parkinson's, and dementia diseases [1–4].

The fatty acid-binding proteins-3 (FABP3) is a small cytoplasmic protein released from cardiac myocytes following an ischemic episode. These molecules are involved in active fatty acid metabolism, where it transports fatty acids from the cell membrane to mitochondria for oxidation. A single session of strength exercise was able to elevate the plasma level of these molecules in both groups immediately after and 2 h after the end of exercise (Figure 4(c)). Even though there is not much information associating the level of plasma FABP3 and exercise, some papers have shown that the level of FABP is influenced by exercise, PPAR-*α* agonists, and testosterone and oscillates with a circadian rhythm. In muscle cells, FABP is involved in the uptake of fatty acids and their subsequent transport towards the mitochondrial *β*-oxidation system [62].

Follistatin-like-1 (FLST1) is a cytokine with diverse actions in the maintenance of cardiac homeostasis and remodeling. Follistatin-like-1 (Fstl1) is a secreted glycoprotein expressed in the adult heart and is induced in response to injurious conditions that promote myocardial hypertrophy and heart failure [63]. Here, the basal levels of FLST1 between TG and UTG present a statistical difference (Figure 4(d)). There is research showing that exercise can induce the elevation of FLST1 in circulation, depending on the type of exercise and the status of the group [63]. Here, the single strength training session elevated the plasma concentration of these molecules immediately after the end of exercise, but only in TG. The plasmatic level of osteonectin, that is, glycoprotein, which plays a vital role in bone mineralization, was not changed. The level of IL-15 that is a cytokine, which regulates the activation and proliferation of T and natural killer (NK) cells, was not affected by the single session of strength training.

Together, these results show that the strength training session is enough stimuli to change physiological parameters such as HR, RPE, VAS, and lactate levels. This exercise protocol also induces changes in the counting of white blood cells, especially neutrophils, lymphocytes, and monocytes. In addition, a single session of strength training can modulate some important myokines such as Apelin, FABP3, and FLST1.

## 5. Conclusion

According to these results, a single session of strength exercise protocol could change the physiological and immunological parameters and stimulate the production and release of important myokines. The acute strength training session induces the elevation of the heart rate, RPE, VAS, lactate, and CK. This protocol also changed the number of white blood cells (neutrophils, lymphocytes, and monocytes) and myokines such as IL-6, BDNF, and FABP3 myokines. In this sense, the strength training session is able to modulate some aspects of immune systems.

### 5.1. Future Perspectives

There is a straight relationship between exercise and the immune system. Exercise may modulate the immune system response acutely and chronically. Nevertheless, physical exercise also induces the release of myokines in the blood circulation. The physiological function of myokines produced by the skeletal muscle is to protect and improve the functionality of many organs. Furthermore, there is convincing evidence that factors secreted by the skeletal muscle act as endocrine signaling mediators and are involved in the beneficial effects of exercise on almost all cell types and organs. From now on, it is important to discover the role of these molecules in the systems and the organs of the body and investigate the difference between physical exercise protocols in the levels of these myokines.

## Figures and Tables

**Table 1:** List of foods offered and their macronutrient values.

Food	Amount	Caloric value	Protein	Carbohydrate	Total fat
Grape juice	200 g	123 kcal	0.6 g	30.2 g	0 g
Orange	90 g	42.3 kcal	0.8 g	10.5 g	0.1 g
Low-fat yogurt	70 g	39.2 kcal	4.0 g	5.3 g	0.1 g
Granola	20 g	77.6 kcal	1.9 g	14.7 g	1.2 g
Whole bread	44 g	110 kcal	4.1 g	20.6 g	0 g
Turkey breast	32 g	44.8 kcal	9.6 g	0 g	0.3 g
Ricotta	20 g	34.8 kcal	2.2 g	0.6 g	2.6 g
Lettuce	10 g	1.3 kcal	0.1 g	0.2 g	0 g
Tomato	30 g	6.1 kcal	0.2 g	1.5 g	0 g
Total	511 g	459 kcal	23.2 g (20.6%)	80.2 g (71%)	4.1 g (8.3%)

**Table 2:** The characterization of the volunteers. This table shows the absolute values and means of each volunteer for weight (kg), height (cm), percentage of body fat (% BF), and age (years) (*p* = 0.05). UTG presented 24.5 ± 2.8 of age, 74.8 ± 14.2 (kg) of body weight, 175.1 ± 8.4 of height (cm), and 15.0 ± 8.7 of percentage of body fat (BF). TG had 26.6 ± 1.3 of age (years), 72.2 ± 3.8 (kg) of body weight, 173.5 ± 7.7 of height (cm), and 9.8 ± 2.9 of percentage of body fat.

Volunteers	Weight (kg)	Height (cm)	% BF	Age	BMI
Untrained (UTG)					
V1G1	60.7	164	16.7	30	22.5
V2G1	82.5	168	30.5	25	29.2
V3G1	63.5	187	4.5	22	18.1
V4G1	68.6	179	8.2	27	21.4
V5G1	55.8	168	4.4	22	19.7
V6G1	66.8	167	5.5	21	23.8
V7G1	83	185	15.2	25	24.2
V8G1	85.4	177	22.8	27	27.1
V9G1	75.5	169	16.9	21	26.4
V10G1	106	186	24.6	25	30.7
Mean	74.8	175.1	15.0	24.5	24.3
SD	14.2	8.4	8.7	2.8	3.8
Trained (TG)					
V1G2	70	167	13	27	25.1
V2G2	74.4	181	7.7	24	22.7
V3G2	74.7	162	13.3	26	28.4
V4G2	77.4	180	6.3	29	23.8
V5G2	70.4	170	7.2	28	24.3
V6G2	68	174	6.9	26	22.4
V7G2	66	162	12	27	25.1
V8G2	77.8	186	10	26	22.4
V9G2	69.2	175	7.6	26	22.6
V10G2	74.4	178	14.1	27	23.4
Mean	72.2	173	9.8	26.6	24.1
SD	3.8	7.7	2.9	1.3	1.8

## Data Availability

The data used to support the findings of this study are available from the corresponding author upon request.

## References

[1] Pedersen B. K., Saltin B. (2015). Exercise as medicine – evidence for prescribing exercise as therapy in 26 different chronic diseases. *Scandinavian Journal of Medicine & Science in Sports*.

[2] Pedersen B. K., Febbraio M. A. (2008). Muscle as an endocrine organ: focus on muscle-derived interleukin-6. *Physiological Reviews*.

[3] Pedersen B. K., Febbraio M. A. (2012). Muscles, exercise and obesity: skeletal muscle as a secretory organ. *Nature Reviews Endocrinology*.

[4] Gomes E. C., Silva A. N., Oliveira M. R. d. (2012). Oxidants, antioxidants, and the beneficial roles of exercise-induced production of reactive species. *Oxidative Medicine and Cellular Longevity*.

[5] Lucas da Nobrega A. C. (2005). The subacute effects of exercise: concept, characteristics, and clinical implications. *Exercise and Sport Sciences Reviews*.

[6] Peake J. M., Nosaka K., Suzuki K. (2006). Characterization of inflammatory responses to eccentric exercise in humans. *Exercise Immunology Review*.

[7] Smith L. L. (2004). Tissue trauma: the underlying cause of overtraining syndrome?. *Journal of Strength and Conditioning Research*.

[8] Tidball J. G. (2005). Inflammatory processes in muscle injury and repair. *American Journal of Physiology-Regulatory, Integrative and Comparative Physiology*.

[9] Smith C., Kruger M. J., Smith R. M., Myburgh K. H. (2008). The inflammatory response to skeletal muscle injury: illuminating complexities. *Sports Medicine*.

[10] Cheung K., Hume P. A., Maxwell L. (2003). Delayed onset muscle soreness: treatment strategies and performance factors. *Sports Medicine*.

[11] Bloomer R. J., Goldfarb A. H., Wideman L., McKenzie M. J., Consitt L. A. (2005). Effects of acute aerobic and anaerobic exercise on blood markers of oxidative stress. *The Journal of Strength & Conditioning Research*.

[12] Bloomer R. J., Falvo M. J., Fry A. C., Schilling B. K., Smith W. A., MOORE C. H. R. I. S. T. O. P. H. E. R. A. (2006). Oxidative stress response in trained men following repeated squats or sprints. *Medicine & Science in Sports & Exercise*.

[13] Bloomer R. J. (2007). The role of nutritional supplements in the prevention and treatment of resistance exercise-induced skeletal muscle injury. *Sports Medicine*.

[14] Bloomer R. J., Schilling B. K., Karlage R. E., Ledoux M. S., Pfeiffer R. F., Callegari J. (2008). Effect of resistance training on blood oxidative stress in Parkinson disease. *Medicine and Science in Sports and Exercise*.

[15] Kraemer W. J., Ratamess N. A. (2004). Fundamentals of resistance training: progression and exercise prescription. *Medicine & Science in Sports & Exercise*.

[16] Franchi M. V., Reeves N. D., Narici M. V. (2017). Skeletal muscle remodeling in response to eccentric vs. concentric loading: morphological, molecular, and metabolic adaptations. *Frontiers in Physiology*.

[17] Perjés Á., Skoumal R., Tenhunen O. (2014). Apelin increases cardiac contractility via protein kinase C*ε*- and extracellular signal-regulated kinase-dependent mechanisms. *PLoS One*.

[18] Schnyder S., Handschin C. (2015). Skeletal muscle as an endocrine organ: PGC-1*α*, myokines and exercise. *Bone*.

[19] Lammers G., Poelkens F., van Duijnhoven N. T. L. (2012). Expression of genes involved in fatty acid transport and insulin signaling is altered by physical inactivity and exercise training in human skeletal muscle. *American Journal of Physiology-Endocrinology and Metabolism*.

[20] Zouein F. A., de Castro Bras L. E., da Costa D. V., Lindsey M. L., Kurdi M., Booz G. W. (2013). Heart failure with preserved ejection fraction: emerging drug strategies. *Journal of Cardiovascular Pharmacology*.

[21] Rosset E. M., Bradshaw A. D. (2016). SPARC/osteonectin in mineralized tissue. *Matrix Biology*.

[22] Rinnov A., Yfanti C., Nielsen S. (2014). Endurance training enhances skeletal muscle interleukin-15 in human male subjects. *Endocrine*.

[23] Borg G. A. V. (1982). Psychophysical bases of perceived exertion. *Medicine & Science in Sports & Exercise*.

[24] Robertson R. J., Goss F. L., Rutkowski J. (2003). Concurrent validation of the OMNI perceived exertion scale for resistance exercise. *Medicine & Science in Sports & Exercise*.

[25] Wilson R. C., Jones P. W. (1989). A comparison of the visual analogue scale and modified Borg scale for the measurement of dyspnoea during exercise. *Clinical Science*.

[26] Cyrino E. S., Zucas S. M. (1999). Influência da ingestão de carboidratos sobre o desempenho físico. *Revista da educacao fisica/UEM*.

[27] Lambert C. P., Frank L. L., Evans W. J. (2004). Macronutrient considerations for the sport of bodybuilding. *Sports Medicine*.

[28] Hernandez A. J., Nahas R. M. (2009). Diretriz da Sociedade Brasileira de Medicina do Esporte: Modificações dietéticas, reposição hídrica, suplementos alimentares e drogas: comprovação de ação ergogênica e potenciais riscos para a saúde. *Revista Brasileira Medicina do Esporte*.

[29] Ziegel E. R., Littell R., Milliken G., Stroup W., Wolfinger R. (1997). SAS® System for mixed models. *Technometrics*.

[30] Hinkelmann K., Kempthorne O. (2008). *Design and Analysis of Experiments*.

[31] Bogdanis G. C. (2012). Effects of physical activity and inactivity on muscle fatigue. *Frontiers in Physiology*.

[32] Walsh N. P., Gleeson M., Shephard R. J. (2011). Position statement. Part one: immune function and exercise. *Exercise Immunology Review*.

[33] Walsh N. P., Gleeson M., Pyne D. B. (2011). Position statement part two: maintaining immune health. *Exercise Immunology Review*.

[34] Kakanis M. W., Peake J. M., Brenu E. W. (2010). The open window of susceptibility to infection after acute exercise in healthy young male elite athletes. *Exercise Immunology Review*.

[35] Tossige-Gomes R., Costa K. B., Ottone V. d. O., Magalhães F. d. C., Amorim F. T., Rocha-Vieira E. (2016). Lymphocyte redox imbalance and reduced proliferation after a single session of high intensity interval exercise. *PLoS One*.

[36] Luk H. Y., McKenzie A. L., Duplanty A. A. (2016). Leukocyte subset changes in response to a 164-km road cycle ride in a hot environment. *International Journal of Exercise Science*.

[37] Strömberg A., Rullman E., Jansson E., Gustafsson T. (2017). Exercise-induced upregulation of endothelial adhesion molecules in human skeletal muscle and number of circulating cells with remodeling properties. *Journal of Applied Physiology*.

[38] Peake J. M., Suzuki K., Wilson G. (2005). Exercise-induced muscle damage, plasma cytokines, and markers of neutrophil activation. *Medicine and Science in Sports and Exercise*.

[39] Neves P. R. D. S., Tenório T. R. D. S., Lins T. A. (2015). Acute effects of high- and low-intensity exercise bouts on leukocyte counts. *Journal of Exercise Science & Fitness*.

[40] Kim H.-K., Konishi M., Takahashi M. (2015). Effects of acute endurance exercise performed in the morning and evening on inflammatory cytokine and metabolic hormone responses. *PLoS One*.

[41] Siedlik J. A., Deckert J. A., Benedict S. H. (2017). T cell activation and proliferation following acute exercise in human subjects is altered by storage conditions and mitogen selection. *Journal of Immunological Methods*.

[42] Nunes-Silva A., Moreira J. M., Freitas-Lima L. C. (2018). Intense aerobic exercise modifies leucocytes number, lymphocyte subpopulation and cytokine levels in peripheral blood. *Gazzetta Medica Italiana Archivo per le Scienze Mediche*.

[43] Santos V. C., Sierra A. P. R., Oliveira R. (2016). Marathon race affects neutrophil surface molecules: role of inflammatory mediators. *PLoS One*.

[44] Žákovská A., Knechtle B., Chlíbková D., Miličková M., Rosemann T., Nikolaidis P. T. (2017). The effect of a 100-km ultra-marathon under freezing conditions on selected immunological and hematological parameters. *Frontiers in Physiology*.

[45] Sakelliou A., Fatouros I. G., Athanailidis I. (2016). Evidence of a redox-dependent regulation of immune responses to exercise-induced inflammation. *Oxidative Medicine and Cellular Longevity*.

[46] Santos V. C., Procida I. R., Zicolau E. A. A. (2013). Effect of regular exercise on leukocyte function in young and middle-aged women. *Journal of Exercise Science & Fitness*.

[47] Natale V. M., Brenner I. K., Moldoveanu A. I., Vasiliou P., Shek P., Shephard R. J. (2003). Effects of three different types of exercise on blood leukocyte count during and following exercise. *Revista Paulista de Medicina*.

[48] Nieman D. C., Pedersen B. K. (1999). *Exercise and immune function*. *Sports Medicine*.

[49] Nunes-Silva A., Bernardes P. T. T., Rezende B. M. (2014). Treadmill exercise induces neutrophil recruitment into muscle tissue in a reactive oxygen species-dependent manner. An intravital microscopy study. *PLoS One*.

[50] Forbes S. J., Rosenthal N. (2014). Preparing the ground for tissue regeneration: from mechanism to therapy. *Nature Medicine*.

[51] Tidball J. G. (2017). Regulation of muscle growth and regeneration by the immune system. *Nature Reviews Immunology*.

[52] Cannon J., Orencole S., Fielding R. (1990). Acute phase response in exercise: interaction of age and vitamin E on neutrophils and muscle enzyme release. *The American Journal of Physiology*.

[53] Cannon J. G., Fiatarone M. A., Fielding R. A., Evans W. J. (1994). Aging and stress-induced changes in complement activation and neutrophil mobilization. *Journal of Applied Physiology*.

[54] Pizza F. X., Mitchell J. B., Davis B. H., Starling R. D., Holtz R. W., Bigelow N. (1995). Exercise induced muscle damage: effect on circulating leukocyte and lymphocyte subsets. *Medicine and Science in Sports and Exercise*.

[55] Malm C., Nyberg P., Engström M. (2000). Immunological changes in human skeletal muscle and blood after eccentric exercise and multiple biopsies. *The Journal of Physiology*.

[56] Pizza F. X., Davis B. H., Henrickson S. D. (1996). Adaptation to eccentric exercise: effect on CD64 and CD11b/CD18 expression. *Journal of Applied Physiology*.

[57] Pizza F., Cavender D., Stockard A., Baylies H., Beighle A. (1999). Anti-inflammatory doses of ibuprofen: effect on neutrophils and exercise-induced muscle injury. *International Journal of Sports Medicine*.

[58] Pizza F. X., Baylies H., Mitchell J. B. (2001). Adaptation to eccentric exercise: neutrophils and E-selectin during early recovery. *Canadian Journal of Applied Physiology*.

[59] Saxton J. M., Claxton D., Winter E., Pockley A. G. (2003). Peripheral blood leucocyte functional responses to acute eccentric exercise in humans are influenced by systemic stress, but not by exercise-induced muscle damage. *Clinical Science*.

[60] Peake J. M., Della Gatta P., Suzuki K., Nieman D. C. (2015). Cytokine expression and secretion by skeletal muscle cells: regulatory mechanisms and exercise effects. *Exercise Immunology Review*.

[61] Moldoveanu A. I., Shephard R. J., Shek P. N. (2001). The cytokine response to physical activity and training. *Sports Medicine*.

[62] Furuhashi M., Hotamisligil G. S. (2008). Fatty acid-binding proteins: role in metabolic diseases and potential as drug targets. *Nature Reviews Drug Discovery*.

[63] Nam J., Perera P., Gordon R. (2015). Follistatin-like 3 is a mediator of exercise-driven bone formation and strengthening. *Bone*.

